# Ex vivo evaluation of corneal filler injection for enhancement after small incision lenticule extraction

**DOI:** 10.1038/s41598-025-22188-8

**Published:** 2025-10-20

**Authors:** Lara Buhl, Maron Dolling, Stefan Kassumeh, Siegfried Priglinger, R. Rox Anderson, Mark Bischoff, Reginald Birngruber

**Affiliations:** 1 Wellman Center for Photomedicine, Massachusetts General Hospital, Boston, MA USA; 2 Department of Ophthalmology, University Hospital, LMU Munich, Mathildenstr. 8, 80336 Munich, Germany; 3 Institute of Biomedical Optics, University of Luebeck, Luebeck, Germany; 4 Corporate Research and Technology, Carl Zeiss AG, Jena, Germany

**Keywords:** Refractive surgery, SMILE, Corneal filler, Enhancement methods, Translational research, Refractive errors, Optics and photonics, Applied mathematics

## Abstract

To investigate the feasibility of transparent corneal filler injection for enhancement after myopic Small Incision Lenticule Extraction (SMILE) overcorrection in an ex vivo eye model. Myopic SMILE procedure with an anticipated correction of -5.6 dpt was performed in 46 whole porcine eyes ex vivo. A hyaluronic acid filler was injected into the SMILE interface through the incision, which was subsequently sealed with fibrin glue. Three-dimensional optical coherence tomography (OCT) was acquired pre- and postoperatively, assessing the central filler thickness and refractive power changes. Based on the central pocket thickness and radius, the refractive power change after filler injection was calculated using the well-known Munnerlyn formula. The filler volume (0.4 to 1.7 µl) correlated linearly with the central pocket thickness (Pearson’s r = 0.90, p < 0.0001; R² = 0.96). Based on the filler volume and central pocket thickness, a filler radius was calculated as 2.77 mm. According to the Munnerlyn formula, a central pocket thickness change from 30 μm to 148 μm corresponded to a calculated increase in refractive power from 2.9 to 14.5 dpt. The injection of a corneal filler via the primary SMILE-incision into the interface following myopic SMILE is a feasible method to partially reverse the refractive change ex vivo. Further development for clinical use is warranted.

## Introduction

The Small incision lenticule extraction (SMILE) procedure has become an increasingly popular option for correcting refractive error^1,2^. It is considered as effective and safe as standard femtosecond laser-assisted in situ keratomileusis (fs-LASIK)^3,4^. Potential advantages over fs-LASIK are higher postoperative biomechanical stability, reduced dry eye, and no flap-related complications^5^.

Retreatment due to residual refractive error may become necessary after the myopic SMILE procedure in 2.3–4.4% of all cases in the first year after treatment^6–8^. Potential reasons for retreatment include an initial under- or over-correction and refractive regression due to increased postoperative tissue remodeling^6^. Enhancement after significant overcorrection of an initially myopic eye is more challenging as it is practically a hyperopia correction.

Enhancement is more elaborate after SMILE than fs-LASIK, yet several procedures are applicable^9,10^. Nevertheless, all procedures include additional tissue ablation to adjust the corneal curvature, which further weakens the cornea’s biomechanical stability and increases the likelihood of iatrogenic ectasia^11^. In the case of overcorrection after SMILE, additional tissue removal can, in principle, be circumvented by replacing parts of the previously removed tissue. Previous studies showed the feasibility of autologous lenticule implantation for hyperopia in patients^12^. Moreover, allogenic intrastromal lenticule reimplantation was investigated for treating residual refractive error after SMILE in animal studies^13,14^. However, because a certain lenticule thickness for dissection and implantation is required, correcting low residual refractive error may be difficult^9^. Additionally, lenticule implantation is a transplantation procedure adding economic and logistic burden to the clinic.

We previously showed that corneal filler injection is feasible for hyperopia and presbyopia correction ex vivo, demonstrating a volume-dependent linear increase in corneal refractive power after filler injection^15,16^. Thus, corneal filler injection could facilitate a precise and adjustable refractive change. We now applied this approach to treating myopic SMILE overcorrection by injecting a biocompatible, transparent filler material into the SMILE interface. In this study, the SMILE incision was used for filler injection and subsequently sealed with fibrin glue.

## Methods

### Tissue preparation

Whole porcine eyes (VisionTech Inc. Sunnyvale, Texas) were processed within 24 hours (h) after death. For corneal edema reduction, the eyes were kept in phosphate-buffered saline (PBS; Fisher Scientific, Waltham, MA) containing 20% w/v dextran (Millipore Sigma, Burlington, MA) for two hours. The whole eyeballs were subsequently mounted on a customized eye holder. A water column was attached to maintain a physiologic intraocular pressure (IOP) of 20-25mmHg.

### Filler material

A 1% w/v hyaluronic acid solution from *Streptococcus equi* (molecular weight, 2.0–2.4 MDa; MilliporeSigma) was prepared with phosphate-buffered saline and kept at 4 °C for further use.

### Small incision lenticule extraction (SMILE) procedure and filler injection

**Fig. 1:**
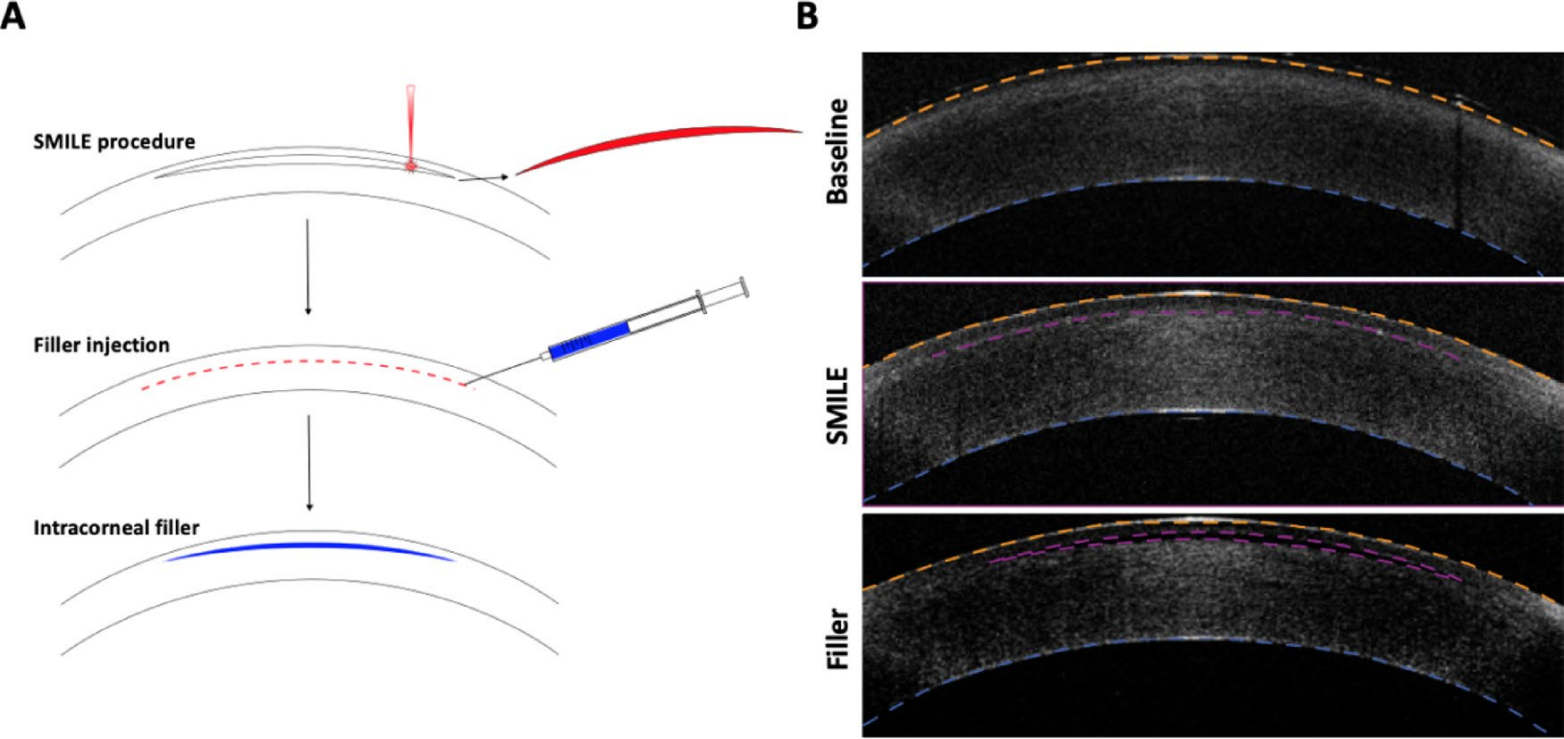
(**A**) Treatment scheme of corneal filler injection for enhancement after SMILE overcorrection. (**B**) Representative OCT B-scans at Baseline, after SMILE treatment, and after filler injection. The anterior curvature is indicated as an orange dotted line, the SMILE interface after laser treatment and after filler injection as a purple dotted line, and the posterior curvature as a blue dotted line.

A step-by-step illustration of the method and corresponding optical coherence tomography images are displayed in Fig. 1A. Before SMILE treatment, a three-dimensional optical coherence tomography (3D-OCT) was performed to acquire a cornea baseline profile. The eye globes underwent a conventional myopic SMILE using a 500-kHz femtosecond ophthalmic surgical laser (VisuMax, Carl Zeiss Meditec AG; Jena, Germany). A myopia correction of −5.6 dpt was performed with no cylinder correction. The central lenticule thickness was 91 μm. The optical zone was 6 mm in diameter with a minimum lenticule thickness of 15 μm. Cap thickness and diameter were 120 μm and 7.2 mm, respectively. The laser pulse energy was 150 nJ, with a pulse repetition rate of 500 kHz. The track and spot distance were both set to 2 μm. Following the laser application, the lenticule was separated from the surrounding stroma using a SMILE spatula (Corza Ophthalmology, Parsippany, NJ, USA) and removed via a 2 mm incision. After the SMILE procedure, another 3D-OCT scan of the cornea was performed to document corneal profile changes. The filler material was then injected using a 30-gauge blunt-ended needle attached to a microliter syringe (Hamilton Company, Virginia, USA) via the SMILE incision. The incision site was then sealed with tissue fibrin glue (Tisseel 2 ml, Baxter, Deefield, IL, USA). 3D-OCT images were acquired directly after filler injection. Figure 1B shows respective 3D-OCT images at baseline, after SMILE, and after filler injection.

### 3D-OCT imaging

A Thorlabs TEL320C1 spectral-domain OCT (SD-OCT) system (Thorlabs, Newton, NJ, USA) with a central wavelength of 1300 nm and axial and lateral resolution of 3.5 μm and 15.6 μm, respectively, was used, covering a field of view (FOV) of 8 × 8 × 3.85 mm with a 91 kHz A-scan rate (~ 2.9 s per volume). A custom-developed calibration algorithm was used to correct the Galvano scanner-introduced distortion using an 8 mm radius spherical phantom (SourcingMap Ltd., San Bruno, CA, USA; quality grade G10).

### Image processing

The tissue surfaces (anterior and posterior cornea and anterior and posterior filler) were extracted, and the beam refraction at all interfaces was calculated using 3D ray tracing based on Snell’s Law of Refraction to correct the interface surfaces. Fit algorithms were used to calculate the radius of the anterior and posterior corneal and filler surfaces (mean square error < 10^−3^ [mm]). For these surface fits, the region of interest (ROI) was defined as 2/3 of the radius of the lenticule (= 4 mm). Pachymetry (central corneal thickness (CCT)) was then calculated as the distance between the anterior and posterior cornea surface in the 1 mm circular center. The central pocket thickness (h) is the distance between the anterior and posterior filler surface. The injected filler volume V was determined postoperatively by calculating the volume between the anterior and posterior filler surfaces (surface integral and voxel count).

### Calculation of the filler-induced refractive change

**Fig. 2:**
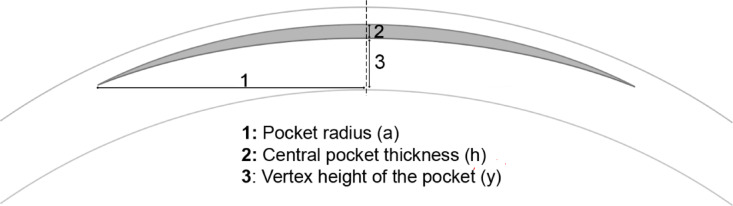
Geometric parameters for calculating the filler-induced refractive change.

Figure 2 shows the pocket geometry and parameters for assessing the filler-induced refractive change. The radius change $\:{\Delta\:}\text{R}\:$of the anterior corneal curvature after filler injection can be calculated using the circular segment equation with the vertex height of the pocket $\:y$, the central pocket thickness $\:h$, and the lateral radius a of the SMILE interface.

1 $$\:{\Delta\:}\text{R}={R}^{*}-\text{R}=\frac{{\text{a}}^{2}\:+\:{(\text{y}+\text{h})}^{2}}{2(\text{y}+\text{h})}-\frac{{\text{a}}^{2}\:+\:{\text{y}}^{2}}{2\text{y}}$$

The formula for the radius change is based on two assumptions: (1) The length of the pocket side-cut is negligible. (2) The filler injection does not induce posterior curvature changes (the anterior curvature is more likely to bend due to the cap loosely laying on top of the pocket).

The change in radius can be converted to the refractive power change $\:{\Delta\:}D\:$using the refractive indices of the air $\:{n}_{1}=1$ and the corneal tissue $\:{n}_{2}\approx\:1.376$:

2 $$\:{\Delta\:}\text{D}=\left({\text{n}}_{2}-{\text{n}}_{1}\right)\left(\frac{1}{{R}^{*}}-\frac{1}{R}\right)\approx\:\left({\text{n}}_{2}-{\text{n}}_{1}\right)\left(\frac{2\left(y+h\right)}{{a}^{2}+{(y+h)}^{2}}-\frac{2y}{{a}^{2}+{y}^{2}}\right)\approx\:\frac{2\left({\text{n}}_{2}-{\text{n}}_{1}\right)\text{h}}{{\text{a}}^{2}}=\frac{0.75\text{h}}{{\text{a}}^{2}}$$

It is worth mentioning that Eqs. [2] and [1] converge to the well-known “Munnerlyn formula” (h = 4a²$\:{\Delta\:}$D/3) when using $\:\left({n}_{2}-{n}_{1}\right)=0.376\approx\:3/8\:$for the air/corneal stroma interface and treating the terms $\:{(y+h)}^{2}$ and $\:{y}^{2}$ both as negligible in comparison to a²^17^.

The relation between the filler volume $\:V$ and the central pocket thickness $\:h$ depends on the radius $\:\text{a}$ of the lenticule.

3 $$\:V=\:\frac{\pi\:}{6}\:\:h\left(3{a}^{2}+{h}^{2}\right)\approx\:\frac{\pi\:}{2}\:\:h{a}^{2}\:{\rm or}:\:\:\text{a}\approx\:\sqrt{\frac{2\text{V}}{{\uppi\:}\text{h}}}$$

*Filler volume* $\:V$ *and central thickness* $\:h$ *were measured after filler injection*,* based on which the radius* $\:\text{a}$ *of the filler can be calculated.*

### Statistical analysis

Statistical analysis was performed using Prism 8 (GraphPad Software, San Diego, CA, USA). Linear regression analysis was used to examine the dependence of corneal refractive power from filler volume and central pocket thickness and respective refractive change. A p-value < 0.05 was considered statistically significant.

## Results

46 porcine eyes were included in this study. In 18 eyes, multiple measurements were performed with different filler volumes, so 68 OCT images were analyzed. After the SMILE procedure, a mean reduction in central corneal thickness of 91.1 ± 14 μm was measured. Injected filler volume was determined by calculating the integral between the anterior and posterior filler curvatures and by voxel-based volumetry. Both volume measurements were consistent with each other (R² = 0.93), indicating a lenticular distribution of the filler. The measured filler volume ranged between 0.4 µl and 1.7 µl. Little to no refractive changes of the posterior curvature were observed upon filler injection (0.08 ± 0.1 dpt, *p* = 0.6).

**Fig. 3:**
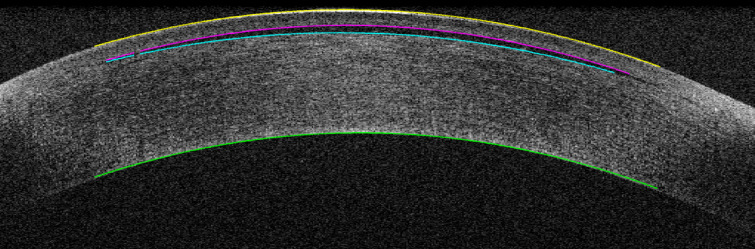
Central OCT B-scan of a pig cornea after filler injection. 0.85 µl were injected inducing a change of h = 72 μm in central corneal thickness (h) resulting in a calculated refractive change of $\:{\Delta\:}\text{D}$ = 7 dpt.

**Fig. 4:**
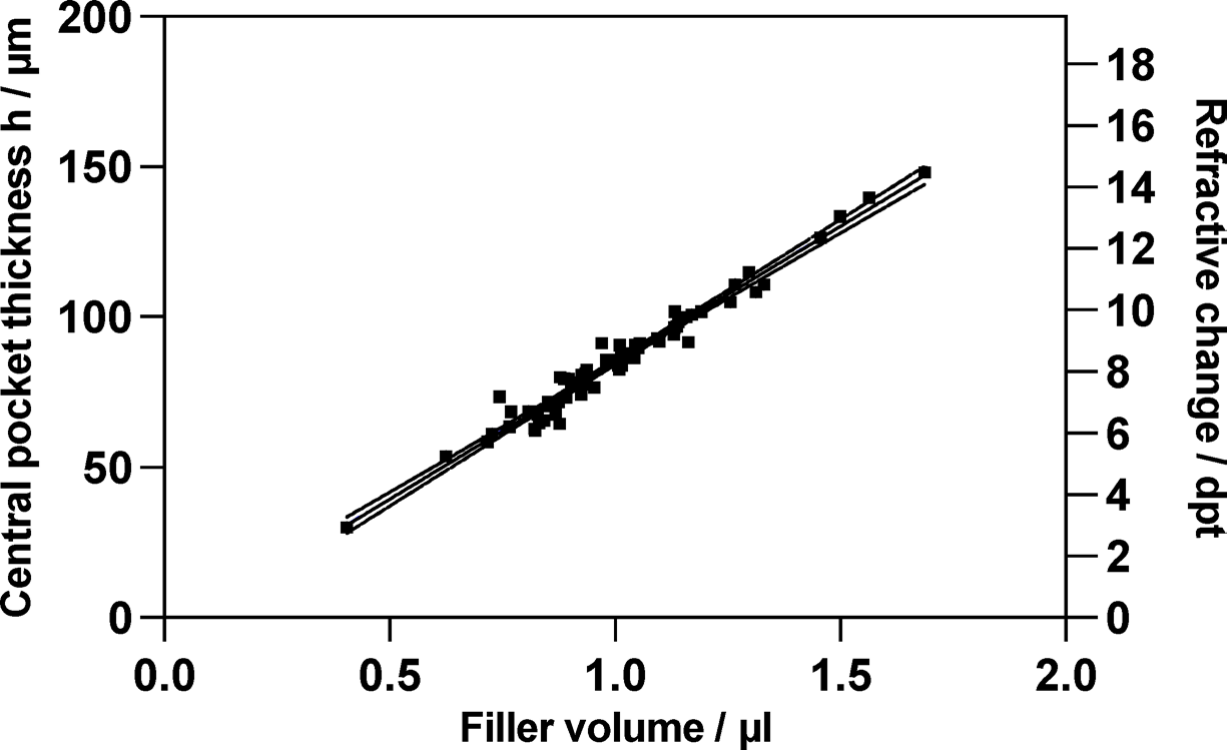
Volume-dependent change in central pocket thickness h and respective corneal refractive power change $\:{\Delta\:}\text{D}$. (Pearson’s *r* = 0.9; *p* < 0.0001).

Figure 3 shows a representative central OCT B-scan of a cornea after injection of 0.85 µl filler, inducing a 72 μm increase in central corneal thickness and a corresponding calculated refractive change of 7 dpt. Figure 4 displays the relationship between filler volume and central pocket thickness, along with the corresponding calculated refractive change of the anterior corneal surface. The filler volume correlated linearly with the central pocket thickness (Pearson’s *r* = 0.90, *p* < 0.0001; R² = 0.96). Based on the measured filler volume and central pocket thickness, a filler radius was calculated as 2.77 mm (see Eq. [3]). This radius was then applied in Eq. [2] to determine the change in corneal refraction depending on the central pocket thickness. Thus, the increase in central pocket thickness from 30 μm to 148 μm corresponded to an increase in refractive power from 2.9 dpt to 14.5 dpt.

## Discussion

In the present pilot study, we evaluated the feasibility of filler injections into the SMILE interface for treating myopic SMILE overcorrection ex vivo. The advantages of this approach are (1) no additional tissue removal, (2) the simple technical feasibility compared to lenticule transplantation, (3) the adjustability of the refractive outcome, and (4) the possibility of complete removal of the filler if necessary.

We used porcine eyes as an experimental model to test the feasibility of increasing the corneal refractive power due to their accessibility and similar biomechanical properties to human eyes. Because central corneal thickness (CCT) measurements are less prone to surgical manipulations than corneal curvature changes, the CCT change after SMILE was assessed, and the refractive change was calculated accordingly. Therefore, the mean reduction in CCT after SMILE matched the expected lenticule thickness of 91 μm with a respective refractive change of − 5.6 dpt. Slight positioning inaccuracies of the eye can explain the variability in CCT change of ± 14 μm before the SMILE procedure.

The refractive power reduction after SMILE can be partially or completely reversed by injecting transparent filler material into the SMILE interface. In addition, the correction can be fine-tuned by adding or removing small amounts of filler material. This is particularly important as previous attempts using additive methods to correct the hyperopia failed primarily due to low predictability^12^. Therefore, a more flexible approach is preferable, allowing further adjustments of the refractive result. As noted above, corneal curvature changes are highly susceptible to surgical manipulations. Therefore, in clinical practice, the final refractive result is assessed as early as one week after surgery. Since this is not experimentally possible, instead of directly measuring the curvature changes, we calculated the increase in refractive change after filler injection into the SMILE interface based on the postoperative central pocket thickness measured by OCT using Eq. [2]. Furthermore, filler pocket radius was calculated as 2.77 mm according to the measured filler volume and central pocket thickness. This value is smaller than the SMILE interface radius (3 mm) or the anterior cap radius (3.6 mm), likely due to the small filler volumes of 0.5 µl to 1.5 µl not fully distributed within the pocket. Equation [2] corresponds to the Munnerlyn formula, which relates central ablation depth to refractive change in myopic LASIK. For this equation to be valid, we verified two assumptions: Firstly, the injected filler expands in the path of least resistance—which, in our case, is anteriorly—due to the SMILE-induced loose cap lying over the stromal bed, with little or no refractive changes of the posterior curvature refraction. This differs from filler use in hyperopia or presbyopia correction, where both corneal curvatures, posterior (P) curvature flattening and anterior (A) curvature steepening, was induced profoundly altering the P/A ratio^15,16^. Secondly, the high correlation between the measured filler volume and the filler thickness (h) confirmed the lenticular-shaped filler distribution, a prerequisite for the ideal refractive correction. Comparable volume estimates obtained from the integral between the anterior and posterior filler surfaces and voxel counts further indicated a lenticular filler shape. With Eq. [2] applied, we observed a linear increase in respective refractive power change with filler injection (Fig. 4), predicting about how much volume is required to achieve the desired change in refraction. Of note, our initial aim was to address methodological limitations using corneal thickness–based refraction predictions (Eq. [2]). Yet, calculating the expected refractive change from tissue removal—using the Munnerlyn formula—is standard in ablative corneal surgery and may be invertibly applied to our approach. However, for this to be valid in vivo, the filler must be fixed in a lenticular shape.

In the present study, we utilized hyaluronic acid (HA) as a filler material that is highly abundant in human connective tissue and widely used in ophthalmic and esthetic surgery^18^. Accidental stromal injection of HA has demonstrated remarkable long-term stability, likely due to its strong water-binding capacity counteracting endothelial dehydration^19,20^. However, its lower refractive index compared with corneal stroma could increase light scattering at the filler/stroma interface, making pure HA most likely suboptimal. Chemically conjugating HA with a higher-index compound, and the option to adjust their ratio, could yield a filler material with both stability and optimal optical properties - high light transmissibility and tunable refractive index. Crosslinking the injected filler material will further enhance its stability and help preserve the lenticular shape upon mechanical stress.

In principle, corneal filler injection can be used for enhancement after SMILE in two ways: Cutting a second intracorneal pocket below the SMILE interface, followed by filler injection, or by direct filler injection into the existing SMILE interface. We chose direct filler injection into the existing SMILE interface as it is technically easier and less invasive. The SMILE incision was closed with fibrin glue after filler injection. In a clinical setting, SMILE retreatment is not performed immediately but at the earliest one week after surgery, when the SMILE incision is re-epithelialized. Therefore, subsequent filler injection would only require partial opening of the 2–3 mm incision and could be more easily reclosed; further in vivo testing of suitable sealing methods will be needed. A possible alternative to fibrin glue for closing the SMILE incision can be photochemical tissue bonding, which has already proven successful in sealing full-thickness corneal lacerations^21^.

Filler titration to achieve the optimal refractive result is a key advantage of our approach over the previously proposed additive procedures, such as hydrogel implants or autologous tissue transplantation. Notably, implementing a reliable preoperative metric to predict filler-induced changes —similarly to the here proposed equation — would be preferable to reduce re-treatment rates. Two scenarios for filler titration are conceivable: (1) Either intraoperative measurement of the refractive outcome during filler injection, for example using real-time aberrometry or intraoperative OCT, or (2) re-treatment one or two weeks after the initial treatment. In the latter, predictions of the expected refractive change can be made based on the initial postoperative outcome (refractive change upon injected filler volume). Crosslinking of the filler material for further stabilization can be performed after reaching the desired refractive outcome. De-crosslinking and subsequent removal of the filler material can be facilitated when only the filler material, but not the filler material to the surrounding stroma would be crosslinked.

In this study, we evaluated a novel conceptual additive approach for SMILE enhancement. Yet, methodical limitations limit the transability into humans at this point. Assessment of filler stability and biocompatibility is not feasible ex vivo due to progressive post-mortem tissue degradation, absence of a systemic immune response, and limited capacity to replicate mechanical stresses—such as blinking or eye rubbing—that are critical for testing filler stability and biocompatibility. Therefore, extensive in vivo studies, particularly with an appropriate filler material, will be required.

In conclusion, we showed that corneal filler injection into the SMILE interface is feasible for correcting myopic SMILE overcorrection ex vivo. Our results show a linear increase in corneal refraction following filler injections into the SMILE interface, allowing precise predictions of the refractive outcome. Yet, in vivo studies are needed to test a suitable filler material and establish a meaningful metric for the volume-dependent increase in corneal refractive power in humans.

## Data Availability

The datasets generated during and/or analysed during the current study are available from the corresponding author on reasonable request.
